# Academic impact and research data utilisation of the clinical practice research datalink: scientometric analyses

**DOI:** 10.1007/s10654-025-01347-1

**Published:** 2026-01-24

**Authors:** Marta Pineda-Moncusí, Maria Rahman, Eleanor L. Axson, Susan Hodgson, Antonella Delmestri

**Affiliations:** 1 Nuffield Department of Orthopaedics, Rheumatology and Musculoskeletal Sciences, Health Data Sciences, University of Oxford, Oxford, UK; 2 Clinical Practice Research Datalink, Medicines and Healthcare Products Regulatory Agency, London, UK

**Keywords:** CPRD, Research output, Scientific production, Electronic health record, EHR

## Abstract

Since its establishment in the late 1980s, the UK Clinical Practice Research Datalink (CPRD) has become one of the most widely utilised data resources in both national and international research. Its value lies in the richness, scale and quality of its routinely collected primary care data, as well as the availability of numerous linkable datasets. This study provides comprehensive scientometric analyses of CPRD-related research output, impact, and data usage from 1988 to 2024. A total of 3779 peer-reviewed publications were identified, and for 98.78% of them, enriched bibliometric metadata were retrieved through Scopus and Web of Science. The UK emerged as the leading contributing country, with the United States and Canada ranking second and third. ‘*McGill University*’ was the most frequently affiliated institution, followed by the ‘*University of Manchester*’ and the ‘*University of Oxford*’, with seven UK universities among the top ten. The three journals most frequently publishing CPRD-based research overall, and since 2020, were ‘*BMJ Open*’, ‘*Pharmacoepidemiology and Drug Safety*’ and ‘*British Journal of General Practice*’. Analyses of primary care data sources utilisation revealed that overall, 86.35% of manuscripts used CPRD GOLD exclusively, 8.39% used both CPRD GOLD and CPRD Aurum, and 4.76% used CPRD Aurum alone, although recent years showed an increased use of CPRD Aurum. Between 2016 and 2024, most articles (80.26%) were associated with CPRD research applications that referenced linked or CPRD algorithm-derived datasets. The three most frequently used were ‘*Hospital Episode Statistics*’ (69.77%), ‘*Small Area Linkages*’ (62.27%) and ‘*Office for National Statistics*’ mortality data (53.28%).

**Supplementary Information:**

The online version contains supplementary material available at 10.1007/s10654-025-01347-1.

## Introduction

In the late 1980s, the United Kingdom (UK) began to routinely collect Electronic Health Records (EHRs) at general practitioner (GP) practices for the purpose of patient and clinic management, followed soon after by data collections at hospitals for their administration. With a large number of patients and longitudinal observations, over time, EHRs have become functional for observational clinical research and epidemiological investigations [1]. Whenever possible, primary care data are linked to secondary care data and to other data sources (e.g. mortality, disease-based or treatment-based registries, and socioeconomic status information), to increase the value over using individual datasets separately [2].

The Clinical Practice Research Datalink (CPRD, https://cprd.com/), previously known as the General Practice Research Database (GPRD) and the Value Added Medical Products (VAMP), is a not-for-profit, cost-recovery UK government research service delivered by the Medicines and Healthcare products Regulatory Agency (MHRA) with support from the National Institute for Health and Care Research (NIHR), as part of the Department of Health and Social Care. CPRD hosts two anonymised observational primary care EHRs: CPRD GOLD [3] established in 1987, and CPRD Aurum launched in October 2017 [4]. CPRD GOLD data are collected from GP practices that use Vision® as their electronic patient record (EPR) software system, while CPRD Aurum data are obtained from those practices that use EMIS Web®. As of December 2024, CPRD GOLD and CPRD Aurum combined contain more than 71 million historical and current patients from the four constituent countries of the UK, of which 19 million are current patients. CPRD GOLD includes historical patients from all of the UK, with current patients from Scotland, Wales and Northern Ireland, while CPRD Aurum comprises patients currently from England.

Practices in England that contribute to these EHRs are offered to participate in CPRD’s anonymised linkage scheme that connects patients’ records across multiple data sources to increase the data’s potential. The linkage is carried out by NHS England as a trusted third party to protect patients’ confidentiality.

Since 2009, several linked data sources have become available to CPRD users, including the Hospital Episode Statistics (HES) (i.e. Admitted Patient Care (APC), Accident & Emergency (A&E), Outpatient (OP), Diagnostic Imaging Dataset (DID)), the Office for National Statistics (ONS) death registration data, the Small Area Linkages (SAL) (i.e. Index of Multiple Deprivation, Rural–Urban Classification, Townsend Deprivation Index, Level Carstairs Index), and the National Cancer Registration and Analysis Service (NCRAS) datasets. During the COVID-19 pandemic, targeted linkages were created and became accessible through CPRD, such as COVID-19 Hospitalisation in England Surveillance System (CHESS), Second Generation Surveillance System (SGSS) COVID-19 positive virology data, COVID-19 Intensive Care National Audit and Research Centre (ICNARC). Some former linkages, such as the HES Patient Reported Outcome Measures (PROMS), Mental Health and Mental Health Services Datasets (MHDS/MHSDS) and the Myocardial Ischaemia National Audit Project (MINAP), are no longer supported. More information about linked data is available at https://www.cprd.com/cprd-linked-data.

To integrate related information across patient care records, CPRD has developed three algorithm-derived datasets based on primary care data, supplemented, where available, by linked secondary care data for the Ethnicity Records (https://www.cprd.com/cprd-algorithm-derived-data). In 2011, the CPRD GOLD Mother-Baby Link, and in 2022 the CPRD Aurum Mother-Baby Link, were launched to link likely mother-baby pairs within CPRD GOLD and CPRD Aurum, respectively [5]. In 2017, the CPRD GOLD Pregnancy Register and in 2021 the CPRD Aurum Pregnancy Register were made available, listing all pregnancy episodes recorded in CPRD GOLD and Aurum, respectively [6–8]. Finally, since 2023 the CPRD GOLD Ethnicity Record and CPRD Aurum Ethnicity Record are available and report a single derived ethnicity category for each patient in CPRD GOLD and CPRD Aurum, respectively [9, 10]. These CPRD Ethnicity Records address the presence in EHRs of multiple and possibly conflicting ethnicity information per patient, as ethnicity is collected as a primary care event and at each secondary care event.

CPRD data sources have been used extensively nationally and internationally for more than thirty-five years. Over time, researchers using CPRD data have produced a substantial body of observational research literature, which in turn has informed pharmacovigilance, regulatory decision-making, clinical practice and healthcare. The CPRD website (https://www.cprd.com/bibliography) reports a remarkable number of research publications that can be used to investigate CPRD’s academic impact, fields’ spread and data sources utilisation. Our study has two main aims. Firstly, to study the CPRD’s academic impact via paper metrics (i.e. longitudinal publication growth, core journals involvement, field range and spread, institutions’ productivity and collaborations, authors’ performance and patterns), and via citation metrics (i.e. citations stratified by year, journal, field, institution and author, and other measures of influence, such as H-index, G-index and M-index). Secondly, to assess CPRD scientific output over time, and stratified by primary care data source (i.e. CPRD GOLD, CPRD Aurum), data linkages (i.e. HES, ONS, SAL, NCRAS, MINAP, MHDS/MHSDS, COVID-19), CPRD algorithm-derived datasets (i.e. CPRD Mother-Baby Link, CPRD Pregnancy Register, CPRD Ethnicity Record), National Institute for Health and Care Excellence (NICE, https://www.nice.org.uk/) guidelines papers, and publications using the CPRD data mapped to the Observational Medical Outcomes Partnership (OMOP) Common Data Model (CDM, https://www.ohdsi.org/data-standardization/).

## Related works

With the first manuscript based on CPRD data dating back to 1988, several bibliometric and scientometric studies have examined CPRD research output, either on its own or in combination and comparison with other UK EHRs. Even if each of these articles focused on some specific objectives, all of them described a clear increase over time in the utilisation of primary care EHRs, confirming the growing role of observational research. Moreover, each study confirmed the major scientific impact of CPRD to inform pharmacovigilance, healthcare and clinical practice in the UK and internationally.

The first paper to investigate the CPRD’s scientific production and academic impact [11] focused on studies using CPRD data published between 1995 and 2009 that were available from the Web of Science (WoS) search engine. The researchers found that the number of CPRD studies grew rapidly, reaching in 2009 a total of 749 manuscripts published in 193 journals across 58 subjects, of which the main ones were ‘Pharmacology and Pharmacy’, ‘General and Internal Medicine’, and ‘Public, Environmental and Occupational Health’. The number of authors was 1251 from 22 countries, of which the UK and the United States (US) were the most active, but less than 1.5% of the authors published nearly 50% of the manuscripts, of which one-third were internationally co-authored.

Another scientometric article studied the research output of the three main UK primary care EHRs: CPRD, The Health Improvement Network (THIN) UK and QResearch [12]. The authors analysed papers published between 1995 and 2015 obtained from the Scopus scientific database and estimated a publication compound annual growth rate of 18.65%. The article reported a total of 1891 papers published in 425 journals, grouped in ten subjects, of which the top three were ‘Medicine’, ‘Biochemistry, genetics and molecular biology’ and ‘Pharmacology, toxicology and pharmaceutics’. The researchers identified 9,385 authors across 29 countries, with the UK (63.56%), the US (29.77%) and Spain (10.15%) as the highest contributors. Eight universities were among the ten most productive institutions, of which the top three were the University of Nottingham (14.06%), Boston University (12.05%), and the Centro Español de Investigación Farmacoepidemiológica (CEIFE, 8.62%). However, when looking at the interquartile range (IQR) of citations, the top three institutions were CEIFE, the University of Pennsylvania and Boston University. Finally, seven of the ten most cited papers were open access, and more than 75% of all papers were published by three or more authors.

Soon after, another bibliometric analysis compared the research output of CPRD, THIN UK and QResearch between 2004 and 2013, and also looked at the CPRD’s publication growth from 1993 to 2013 as a case study [13]. The authors used WoS as a scientific database, and also information on THIN UK provided by University College London (UCL). They found that the literature growth across all three EHRs had been consistent over the 9 years, and that collectively they had produced 1296 publications with CPRD representing the large majority (63.6%), followed by THIN UK (30.4%) and QResearch (5.9%). Because CPRD primary care data at that time only consisted of CPRD GOLD, and THIN UK also derived its data from the same EPR (Vision®), the study concluded that 94% of the UK observational research output was based on data collected by Vision®.

Lastly, using the Scopus scientific database, a scientometric study retrieved research articles from CPRD, THIN UK and QResearch to investigate respiratory conditions, cardiovascular disease and the COVID-19 pandemic [14]. The authors did not compare the contributions of these three EHRs, and overall identified 1222 manuscripts between 2020 and 2022, with 46.24% of the papers related to cardiovascular disease, 30.11% to respiratory conditions and 23.65% to COVID-19. The researchers also explored the distribution of research output up to 2022 for the three main respiratory conditions: asthma, chronic obstructive pulmonary disease (COPD) and interstitial lung disease (ILD). When more of these conditions were investigated in the same article, the study selected the core one and found a steady increase in total publications per year since 2010, with the majority of papers investigating asthma (60.22%), followed by COPD (35.45%) and ILD (4.33%).

## Data and methods

### CPRD research productivity

We started from the CPRD bibliography available at https://www.cprd.com/bibliography, with the aim to include only peer-reviewed journal manuscripts in English, and excluding any publications in other languages, pre-prints, reviews, abstracts, posters, letters or opinions. We downloaded the CPRD bibliography and imported it into a PostgreSQL v. 13 database. We checked all the papers published between 1988 and 2024 against the output of a 14-condition query (available in the Online Resource 3, tab ‘cprd_bibliography_query’) run on the Ovid Medline database of biomedical articles, which we also imported into PostgreSQL. Any discrepancy between the two lists was manually evaluated and the CPRD eligible bibliography was updated. We then calculated CPRD research productivity and its growth over time.

### CPRD scientific impact

To investigate CPRD’s scientific impact, we focused on a combination of established quantitative methods for paper metrics (i.e. core journals’ participation and impact factors, study fields variety, institution and authors’ productivity) and for citation metrics (i.e. citation counts, H-index, G-index and M-index). We intentionally disregarded alternative methods based, for example, on social media posts (e.g. Twitter/X, Facebook, LinkedIn), online engagement (e.g. number of downloads), etc., as these may be considered biased.

To measure CPRD’s scientific impact between 1988 and 2024, we started from the CPRD eligible papers and created their enriched metadata by utilising both Scopus and WoS online scientific databases. We designed a three-step search algorithm, as follows. Step 1: Within these CPRD manuscripts, we identified the papers associated with a Digital Object Identifier (DOI) and searched for them by DOI in Scopus and WoS. Step 2: We searched by title in both databases for those papers that were not found in Step 1 or did not have a DOI. Step 3: For those papers still not retrieved which were associated with a PubMed Identifier (PMID), we searched Scopus and WoS by PMID. We performed data checks at each step and ensured no papers were wrongly retrieved due to errors in the search engine repositories. The order of steps was established on the likelihood for a field to provide a full match: for example, DOIs are supposed to be unique, titles could be duplicated in principle and are much more likely to fail a match due to their longer length, presence of possible special characters and typos, while PMIDs only exist for article indexed in the PubMed database.

At each step we exported from both databases the enhanced metadata for the groups of retrieved papers, and used the comprehensive R package ‘*bibliometrix’* v. 4.3.1 [15] to merge them, excluding duplicates, and analyse the data through R (4.5.1 on RStudio Version 2024.12.0+467). Using ‘*bibliometrix’*, we reported the most frequently used peer-reviewed journals (as number of cumulative CPRD publications), number of single- and multi-authored papers, most productive countries (as number of publications per country in the corresponding author’s affiliation), and most cited papers (as number of cumulative citations as reported by Scopus and WoS metadata). We also reported the number of distinct authors, most recurrent affiliations (sum of each author’s affiliation included in any of the publications), year of first CPRD publication from the top 20 most productive authors (as number of cumulative CPRD publications), and total author’s citations and index metrics (including H-index, G-index and M-index) as reported by Scopus and WoS metadata. Additionally, we reported the evolution of topics of interest using the keywords from authors and Keywords Plus (i.e., from Scopus and WoS). For the most frequently occurring affiliations, we excluded those inadmissible (e.g. ‘A, corresponding author’) and merged those variations that undoubtedly referred to the same affiliation (e.g. ‘IMPERIAL COLL LONDON’, ‘IMPERIAL COLL’, ‘IMPERIAL COLLEGE’).

### CPRD data sources utilisation

Before researchers can start working with CPRD data for a study, they need to submit a research application, known as a protocol. Since June 2021, protocol submission is via CPRD’s Research Data Governance (RDG) process (http://www.cprd.com/research-applications), which replaced the Independent Scientific Advisory Committee (ISAC) for MHRA database research established in 2006. To measure the CPRD data sources utilisation over time, stratified by primary care data source (CPRD GOLD, CPRD Aurum), data linkages (i.e. HES, ONS, SAL, NCRAS, etc.), and CPRD algorithm-derived data (i.e. CPRD Mother-Baby Link, CPRD Pregnancy Registers, CPRD Ethnicity Records), we used the link between each paper and its CPRD protocol, which reports the requested data sources. We assumed that all papers published before 2019 used CPRD GOLD as their primary care data source, since CPRD Aurum was only released in late 2017. For linked and CPRD algorithm-derived data sources, we considered only papers published between 2016 and 2024, as in 2015, CPRD started making protocols publicly available and required authors using CPRD data to report the protocol identifier in their publications. We grouped linked and CPRD algorithm-derived data sources into categories: for example, HES APC, HES A&E, HES OP, HES DID, and HES PROMS were put in the ‘HES’ category. CHESS, ICNARC and SGSS were grouped into the ‘COVID-19’ category. This data source categorisation is available in the Supplementary Material (Appendix 3, tab ‘cprd_datasource_categories’).

### CPRD NICE guidelines papers

NICE is a public body of the UK Department of Health and Social Care dedicated to developing evidence-based recommendations for health and care practitioners on a wide range of topics, including public health, clinical practice and health technologies.

To identify the papers investigating NICE recommendations within the CPRD bibliography, we searched for those papers whose title contained the string “NICE”, and we double-checked those manually.

### CPRD OMOP CDM papers

CDMs are standardised structures for organising heterogeneous data sources generated by diverse healthcare systems (e.g. EHRs, Registries, Claims). By enabling efficient analyses and results comparison across different data sources, CDMs promote international, network-based observational research. In the last two decades, several CDMs have been developed: OMOP, Sentinel, Patient-Centred Clinical Research Network (PCORnet) and Informatics for Integrating Biology and the Bedside (i2b2), among others.

OMOP CDM has been endorsed by different initiatives funded by the Innovative Medicines Initiative (IMI), now known as the Innovative Health Initiative (IHI), and other public– private partnerships. Some of these well-known funded projects are the European Health Data and Evidence Network (EHDEN, https://www.ehden.eu/), the Observational Health Data Sciences and Informatics (OHDSI, https://ohdsi.org/), the Data Analysis and Real World Interrogation Network (DARWIN EU®, https://www.darwin-eu.org/) , and the OPTIMA (https://www.optima-oncology.eu/) and HIPPOCRATES (https://www.hippocrates-imi.eu/) consortia, among others. The OMOP CDM has an open-source, open-science approach, which makes research transparent, reproducible and sustainable, following the FAIR principles for research data and research software to be Findable, Accessible, Interoperable, and Reusable. Reviews have identified in OMOP the CDM most cited [16, 17], and par­ticularly successful in network studies [18, 19].

CPRD data have been transformed to the OMOP CDM by several organisations (e.g. Janssen Research and Development, University of Oxford, US National Institutes of Health, etc.), and currently, CPRD has mapped CPRD Aurum and CPRD Aurum linked to HES APC to the OMOP CDM format.

To identify the papers that used CPRD data transformed to the OMOP CDM, we searched within the CPRD eligible bibliography for those papers that mentioned any of the following strings in the title, abstract or keywords: “Observational Medical Outcomes Partnership”, “OMOP”, “EHDEN”, “OHDSI”, “DARWIN”, “federated analytics” and “network study”. Those papers that matched the latter were further screened manually.

## Results

### CPRD research productivity

**Fig. 1:**
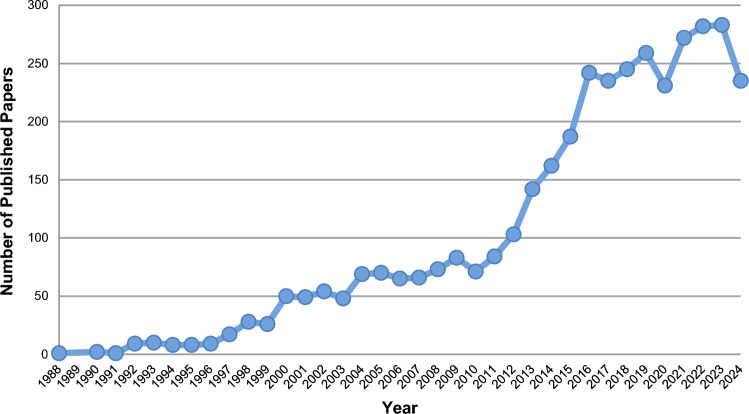
CPRD research productivity in 1988–2024

Starting from the CPRD available bibliography, and cross-checking it against Ovid Medline, we identified 3779 eligible peer-reviewed journal manuscripts published between 1988 and 2024. Figure 1 shows a clear growing trend of CPRD research productivity over time. The maximum number of manuscripts per year was 283 in 2023, with a median [IQR] of 69.5 [23.75–198]. The compound annual growth rate (CAGR) was 16.37%. Numbers are available in the Online Resource 3, tab ‘cprd_research_productivity’.

**Fig. 2:**
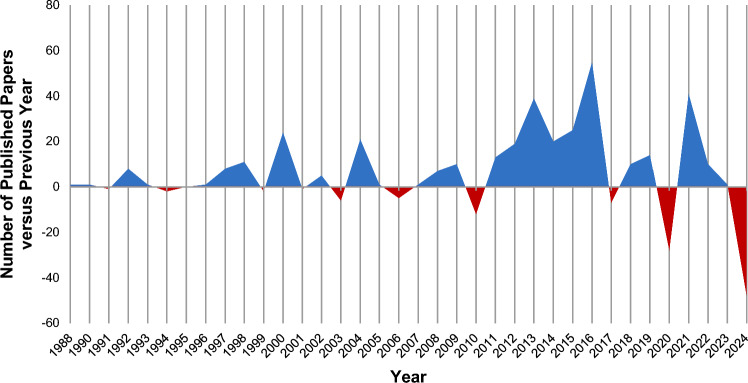
CPRD research growth in 1988–2024

Figure 2 shows that between 1988 and 2024, there were 25 years in which growth increased compared to the previous year, with the highest annual increase recorded in 2016 at 55 units. One year reported no change, and the remaining nine years experienced negative growth, with the most significant decline occurring in 2024 at 48 units. No negative growth occurred in two consecutive years. Numbers are available in the Online Resource 3, tab ‘cprd_research_productivity’.

### CPRD scientific impact

**Fig. 3:**
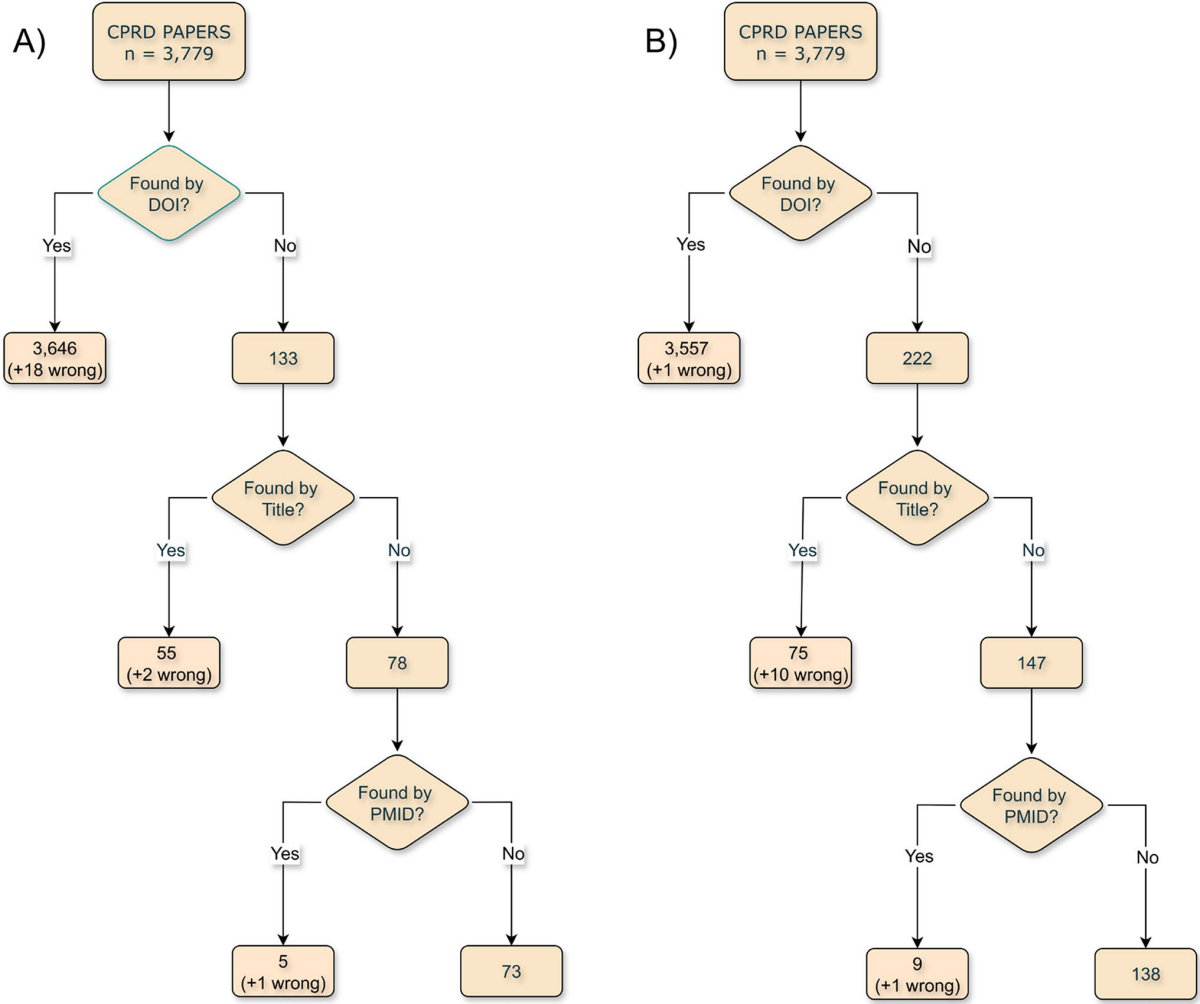
Three-step search algorithm applied to **A** scopus and **B** web of science

Of the 3779 papers included in the CPRD eligible bibliography up to December 2024, we identified 3715 associated with working Digital Object Identifiers (DOIs) and on the 21st of June 2025, we searched for them by DOI in Scopus and WoS. From this group, we retrieved 3646 papers in Scopus and 3557 in WoS. We then searched by title in both databases for those papers that were not found before or did not have a DOI. From this group, we retrieved 55 papers in Scopus and 75 in WoS. Finally, for those papers still not retrieved and associated with a PubMed Identifier (PMID), we searched Scopus and WoS by PMID and retrieved 5 and 9 papers, respectively. We performed data checks at each step and identified 21 papers wrongly retrieved in Scopus and 10 in WoS due to errors in the search engine repositories (i.e. same DOI wrongly associated with multiple publications, papers associated with the wrong PMID, etc.), which we discarded. We also found several misspellings and typos in Scopus and WoS manuscript titles and DOIs, which we corrected. We communicated all these issues to both scientific databases and suggested corrections. Overall, we retrieved 3,733 papers (98.78%): 3706 papers (98.07%) in Scopus and 3641 in WoS (96.35%), with an overlap of 95.63%, and only 46 papers (1.22%) were missing in both databases. Figure 3 below shows the three-step search algorithm applied to (A) Scopus and (B) WoS. The six retrieved files are available in *cprd_biblio_scopus_wos.zip,* as part of the Supplementary Material (Appendix 2).

**Fig. 4:**
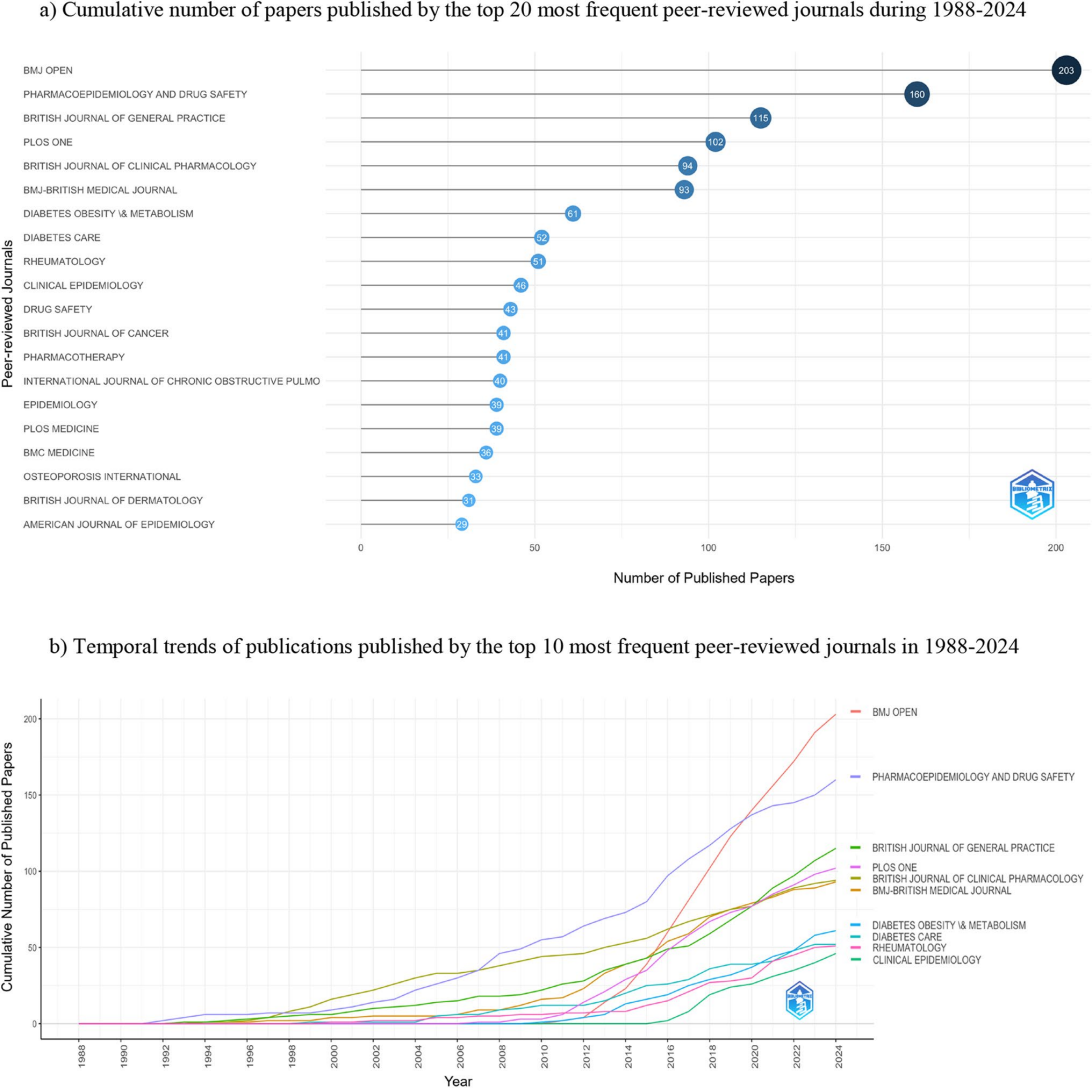
Most frequent peer-reviewed journals using CPRD data in 1988–2024: **a** number of cumulative publications by the top 20 most frequent peer-reviewed journals, and **b** number of cumulative publications over time by the top 10 most frequent peer-reviewed journals

The 3733 papers retrieved from 1988 to 2024 were published in 625 peer-reviewed journals. Until 2019, ‘*Pharmacoepidemiology and Drug Safety*’ was the most frequent journal, with a total of 128 (3.43%) manuscripts. In 2020, ‘*BMJ Open*’ became the most often used journal with 140 (3.75%) papers. By 2024, ‘*BMJ Open*’ reached a total of 203 (5.44%) publications, followed by ‘*Pharmacoepidemiology and Drug Safety*’ with 160 (4.29%) articles. Figure 4a and b reports these results with data available in Online Resource 3, tabs ‘cprd_frequent_journals_overall’ and ‘cprd_top10_journals_overtime’.

**Fig. 5:**
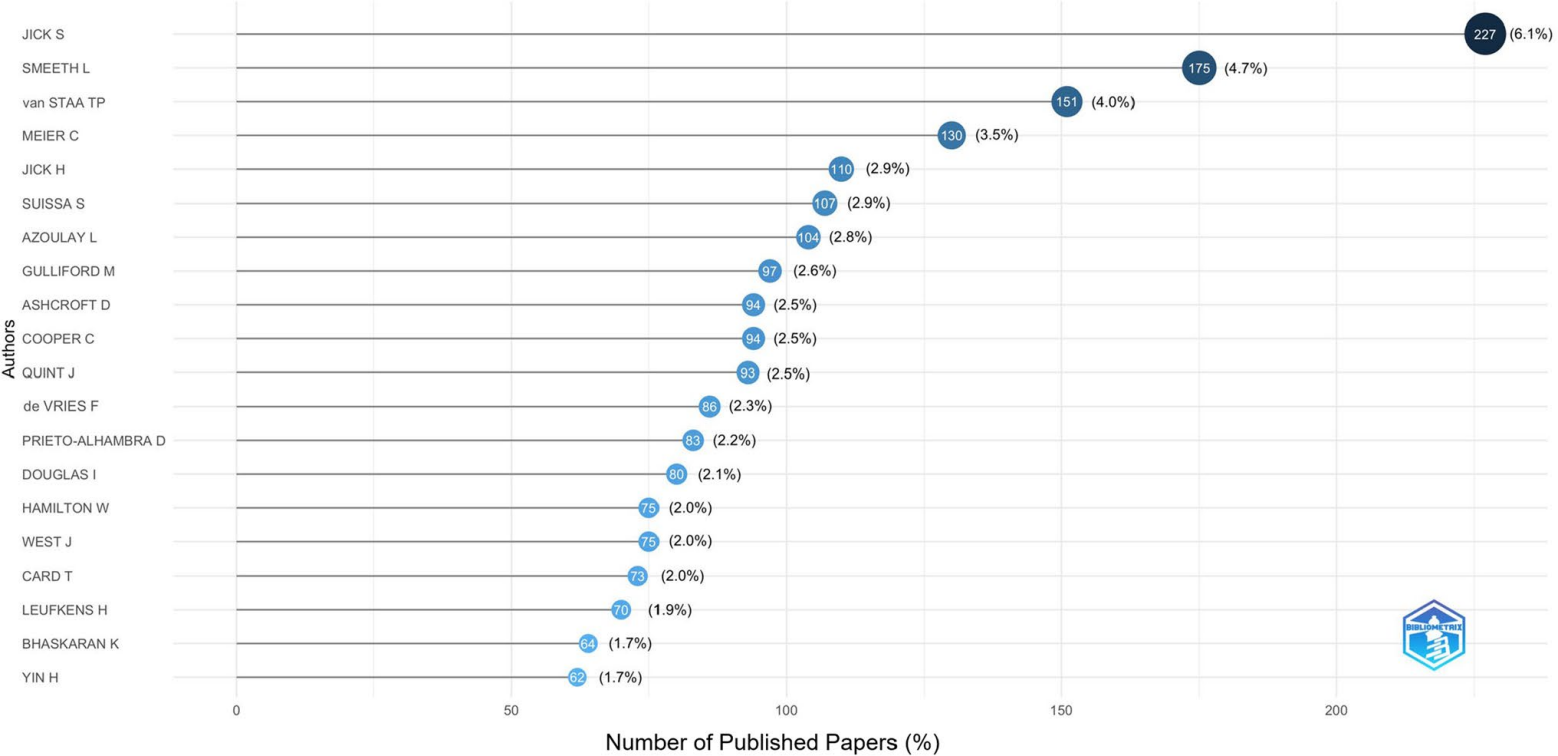
Top 20 most frequently published authors in publications using CPRD data in 1988–2024

We identified 9319 distinct authors (available in the Online Resource 3, tab ‘cprd_authors’) and the top 20 most prolific are displayed in Fig. 5. Citation metrics (median [IQR]) of the overall authors were 1 [1-2] for H-index (maximum value of 59), 1 [1-3] for G-index (maximum value of 114), 0.21 [0.13–0.36] for M-index (maximum value of 2.57), 37 [12–125] of total citations count (maximum value of 14,244). Maximum values in H-index, G-index, M-index and total citations count were observed among the top 20 most prolific authors. These 20 authors had a median [IQR] of 38 [30–48.25] in H-index, 70 [58.25–94] in G-index, 1.65 [1.53–1.94] in M-index, and 5094 [3664–9167] in total citations count. Their first publication was dated between 1990 and 2013 (median [IQR]: 2003 [1997–2008]). The most prolific author, Jick S., has published between 1990 and 2024, with an H-index of 59, G-index of 112, and M-index of 1.6. Data are available in Online Resource 3, tab ‘cprd_citations_top20authors’.

**Fig. 6:**
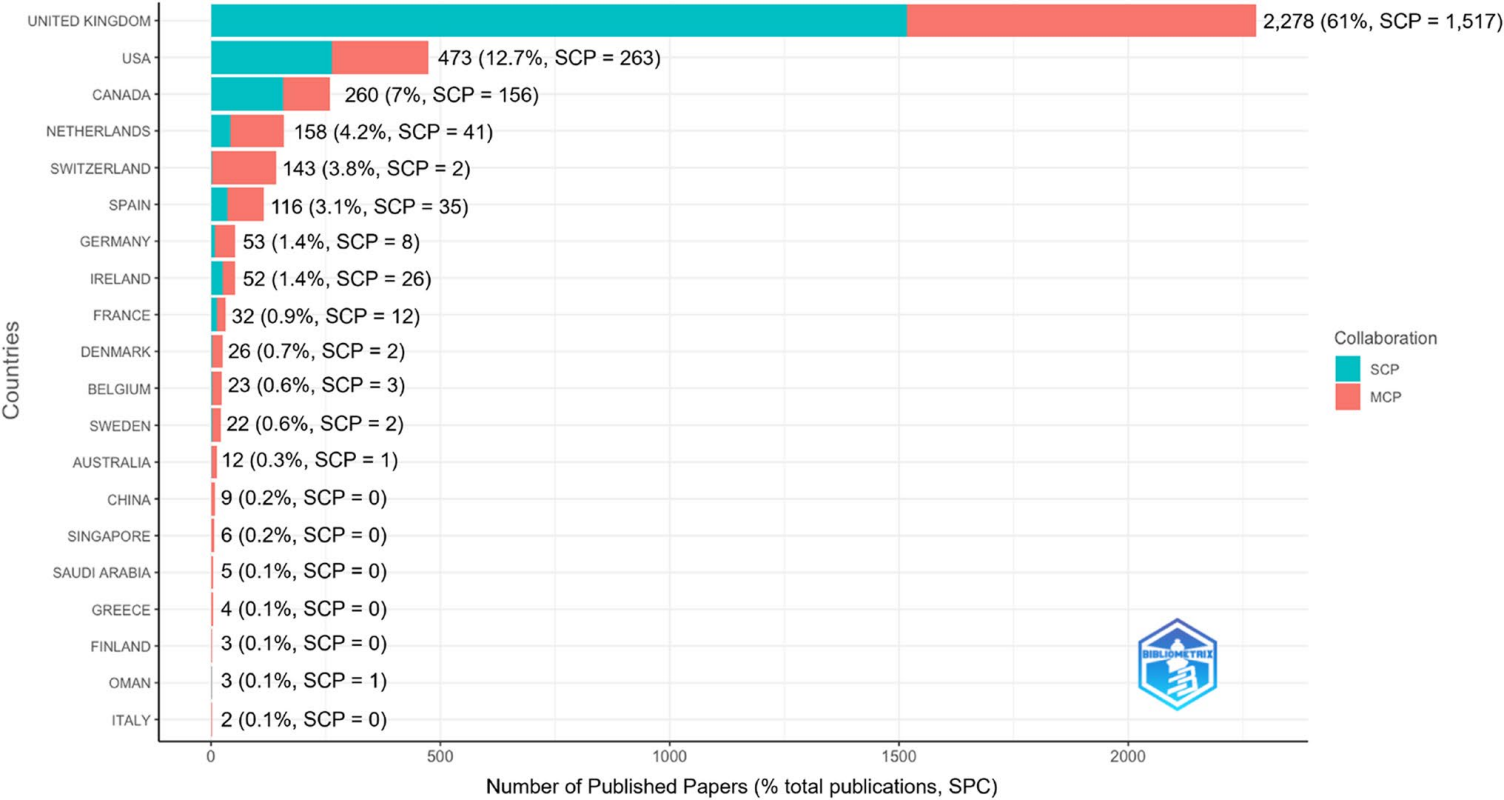
Top 20 most productive countries and the number of published papers using CPRD data in 1988–2024. Single Country Publications (SCP) refer to articles where all co-authors have the same country of affiliation. For Multiple Country Publications (MCP), the main country shown in the Y axis was determined by the corresponding author’s affiliation

Papers had a median of six (IQR: 5–9) co-authors with a maximum of 203, and only 18 publications (from 17 authors) were single-authored. 61.02% (n = 2,278) of the publications included authors from the UK, of which 33.41% (n = 761) involved authors also from other countries. Overall, 43.61% (n = 1,628) of the papers had an international co-authorship (Fig. 6; data available in the Online Resource 3, tab ‘cprd_contributing_countries’).

**Fig. 7:**
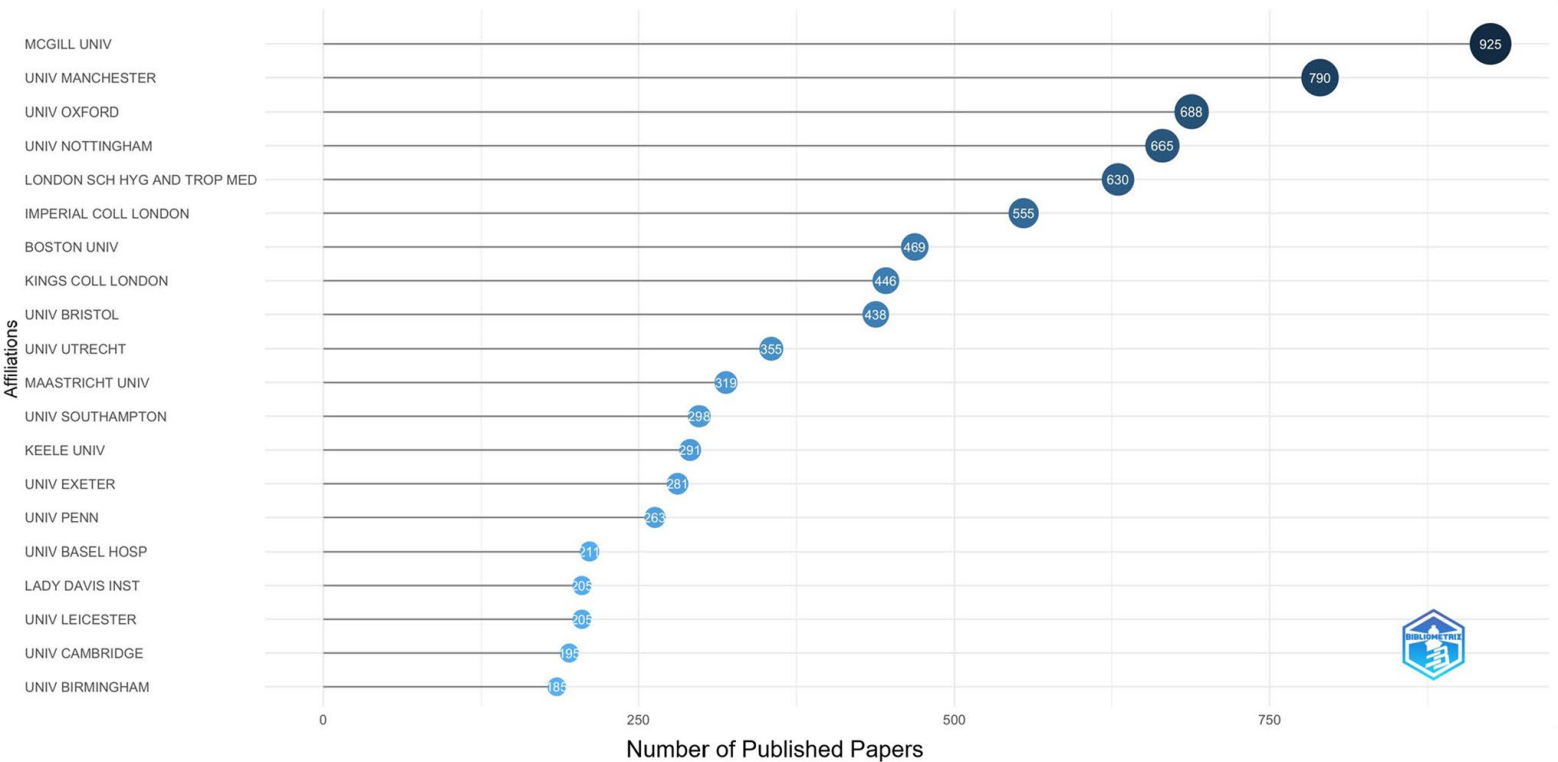
Top 20 most recurrent affiliations of all co-authors of papers using CPRD data in 1988–2024

Figure 7 shows the most recurrently cited authors’ affiliation, which was the Canadian McGill University (n = 925 (4.90%) denoted as ‘MCGILL UNIV’). The second one was the UK University of Manchester (n = 790 (4.19%), denoted as ‘UNIV MANCHESTER’) followed by the UK University of Oxford (n = 688 (3.65%), denoted as ‘UNIV OXFORD’). Data are available in the Online Resource 3, tab ‘cprd_affiliations’. Each author’s affiliation included in any of the publications has been added to the total count, resulting in 18,868 affiliations cited across the 3733 retrieved papers.

**Fig. 8:**
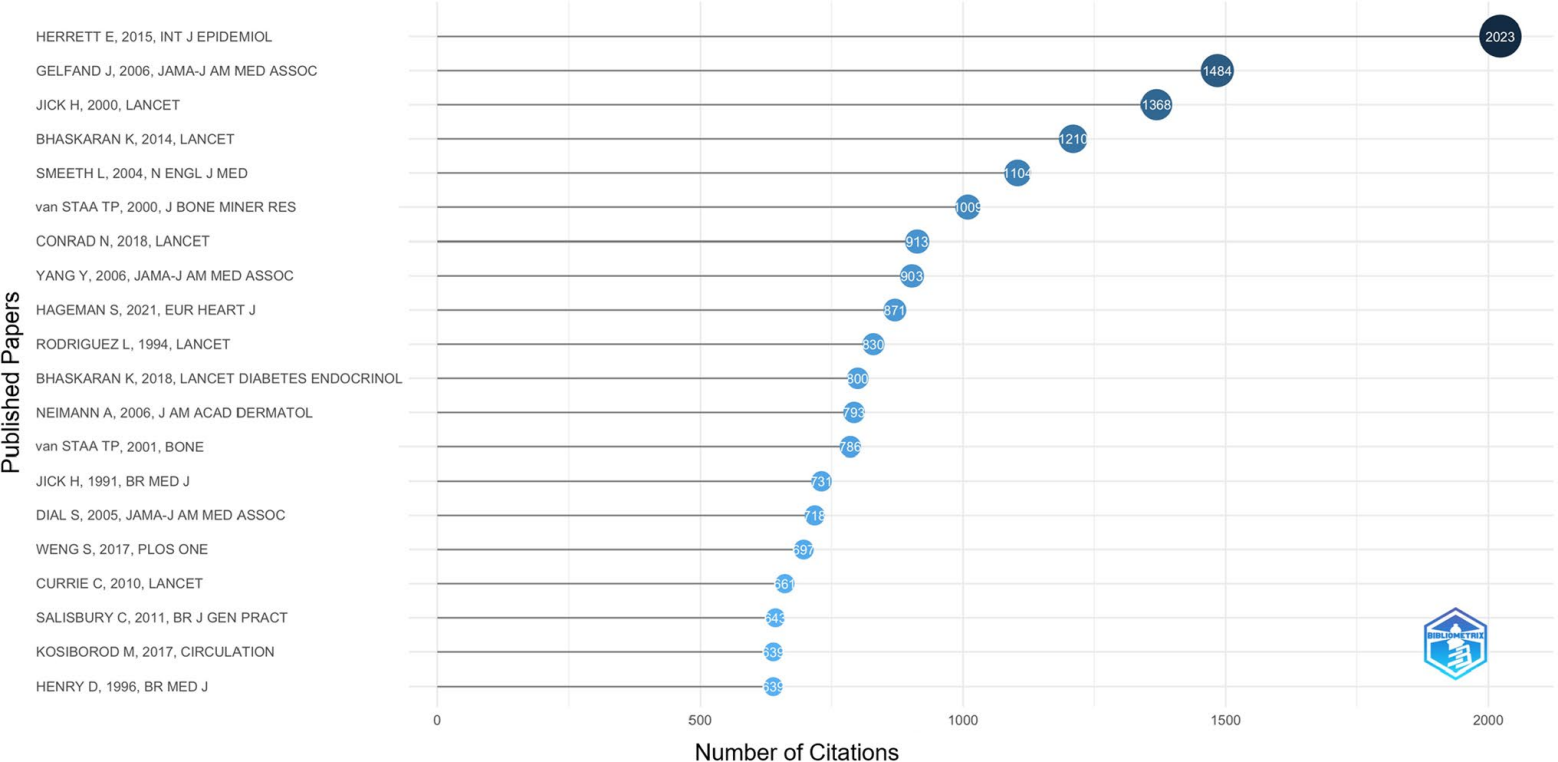
Top 20 most cited papers using CPRD data in 1988–2014

Figure 8 summarises the top 20 most cited papers based on CPRD data (available in the Online Resource 3, tab ‘cprd_papers_citations’). The most cited paper by far, with 2023 citations, was “Data Resource Profile: Clinical Practice Research Datalink (CPRD)” by *Emily Herrett *et al., published in 2015 in the *International Journal of Epidemiology*. The second most cited paper, with 1,484 citations, was “Risk of myocardial infarction in patients with psoriasis” by *Joel M. Gelfand *et al., published in 2006 in *JAMA.* The third, with 1,368 citations, was “Statins and the risk of dementia” by *Hershel Jick *et al., published in 2000 in *The Lancet*.

**Fig. 9:**
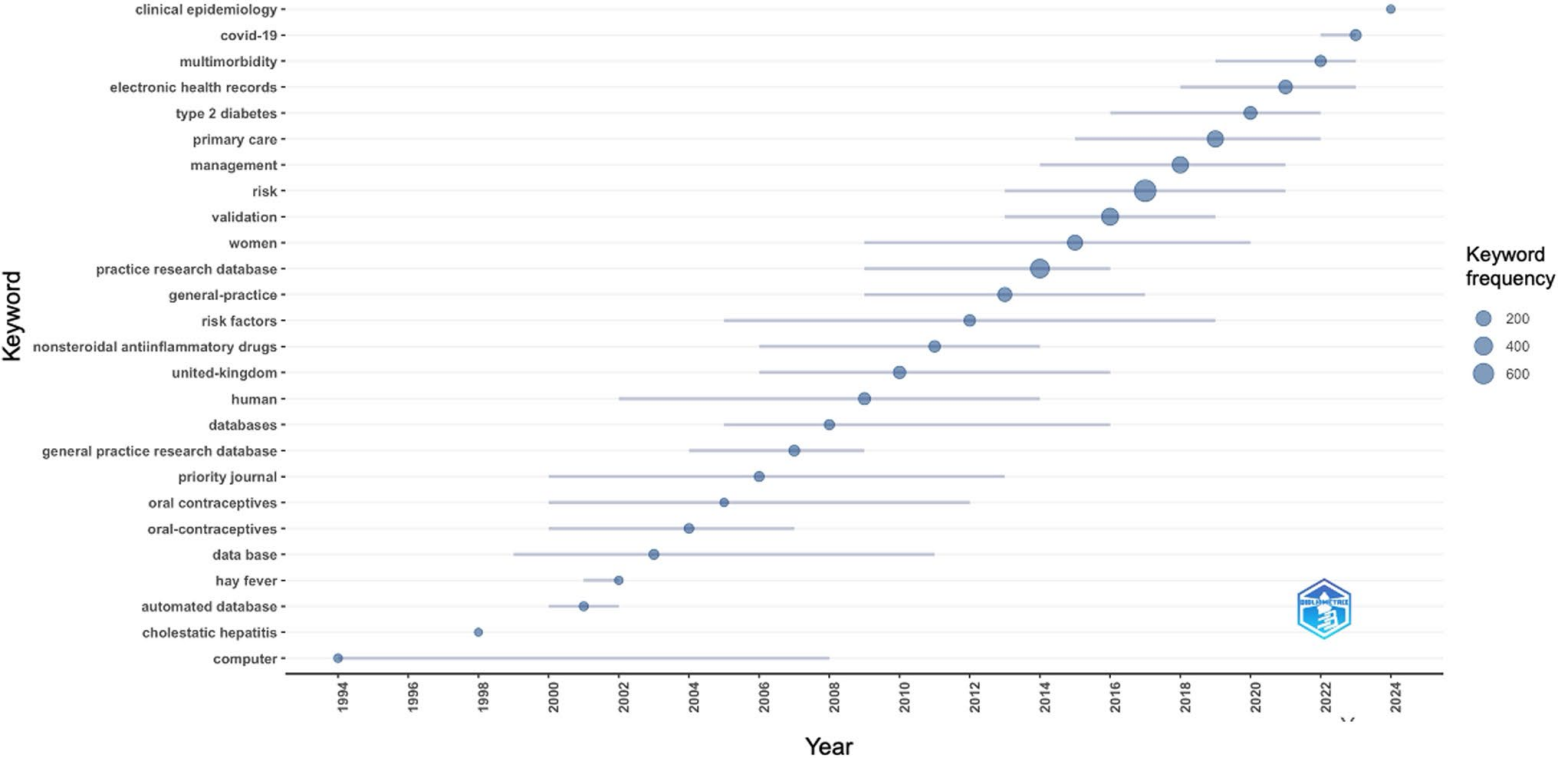
The top one annual prevalent keyword in articles using in CPRD data in 1994–2024, based on Keywords Plus from Scopus and Web of Science

Figure 9 displays the most common paper keyword per year between 1994 and 2024, based on Keywords Plus from Scopus and WoS (data available in the Online Resource 3, tab ‘cprd_annual_top_keyword’). The blue bubbles indicate the annual frequency of a keyword, whilst the blue lines represent the interquartile range of the keyword’s frequency. Words had to be used a minimum of five times to qualify for the annual top (i.e., minimum frequency of five means the keyword was included in five different articles during the same year). We observed that topics evolved over time. The first most frequent word between 1994 and 2000 was ‘computer’, which was used 7 times in 1994. Between 2001 and 2010, it was ‘United-Kingdom’ (used 81 times in 2010), followed by ‘human’ (used 71 times in 2009) and ‘general practice research database’ (used 37 times in 2004). Between 2011 and 2020, the most used keyword was ‘risk’ (used 726 times in 2017), followed by ‘practice research database’ (used 456 times in 2014) and ‘validation’ (used 328 times in 2016). Between 2021 and 2024, the most frequently used keyword was ‘electronic health records’ (used 131 times in 2021), followed by ‘multimorbidity’ (used 46 times in 2022) and ‘COVID-19’ (used 41 times in 2023).

### CPRD data sources utilisation

**Fig. 10:**
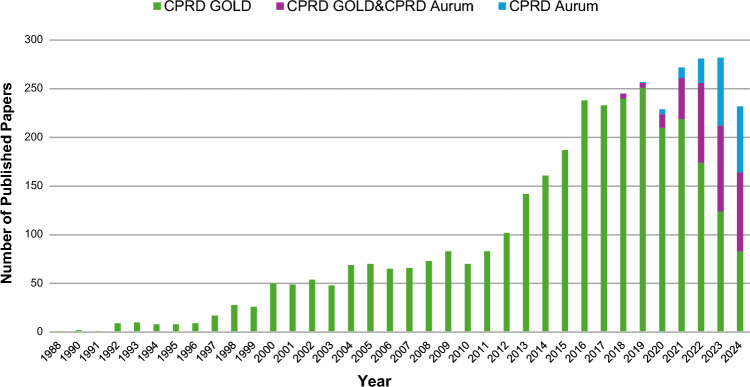
CPRD primary care data source utilisation in 1988–2024

Of the 3779 papers included in the eligible CPRD bibliography up to December 2024, 3263 (86.35%) were based on CPRD GOLD only, 317 (8.39%) on both CPRD GOLD and CPRD Aurum, and 180 (4.76%) on CPRD Aurum only. Four papers were classified manually with respect to the primary care data used, as we could not retrieve their protocol identifier, and they were published when both CPRD GOLD and CPRD Aurum were available. Nineteen papers did not use CPRD data and were categorised manually as ‘Bibliography’ (n = 4), ‘Methodology’ (n = 2), ‘Policy’ (n = 5), ‘Regulatory’ (n = 2), ‘Study protocol’ (n = 3) and ‘Technology’ (n = 3). Category data are available in the Online Resource 3, tab ‘cprd_paper_category’. Figure 10 reports the CPRD primary care data source utilisation of the eligible 3760 papers over time, stratified by CPRD GOLD, CPRD GOLD & CPRD Aurum, and CPRD Aurum. After the CPRD Aurum launch, the exclusive utilisation of CPRD GOLD started decreasing, and the usage of CPRD Aurum increased, especially in conjunction with CPRD GOLD. Data are available in the Online Resource 3, tab ‘cprd_primarycare_utilisation’.

Between 2016 and 2024, we found 2284 papers, fifteen of which did not access CPRD data. For 99.34% of the remaining 2269 manuscripts, we found the associated CPRD protocol identifier, while 15 papers were classified manually regarding linked and CPRD algorithm-derived datasets, as we could not retrieve their CPRD protocol identifier. Of all the 2269 manuscripts that used CPRD data, 80.26% were associated with a CPRD protocol that requested linked or CPRD algorithm-derived datasets.

As the capability of CPRD to provide linked and CPRD algorithm-derived datasets changed over time, the time windows (in years) of the CPRD delivery service are presented in Table 1 stratified by the CPRD primary care data source.

**Table 1:** Time windows of CPRD delivery service of linked datasets and CPRD algorithm-derived datasets, stratified by CPRD primary care data source

	CPRD GOLD Start–end year	CPRD aurum Start–end year
Linked dataset		
HES (any)	2009—present	2017—present
SAL (any)	2009—present	2017—present
ONS	2009—present	2017—present
NCRAS (any)	2009—present	2017—present
MINAP	2009—2017	N/A
MHDS/MHSDS	2017—2021	2017—2021
COVID-19 (any)	2020—2025	2020—2025
CPRD algorithm-derived dataset		
CPRD Mother-Baby Link	2011—present	2022—present
CPRD Pregnancy Register	2017—present	2021—present
CPRD Ethnicity Record	2023—present	2023—present

Table 2 reports the CPRD primary care data, linked datasets and CPRD algorithm-derived datasets stratified by CPRD primary care data source requested in the CPRD approved protocols for this group of articles. The three most used linked datasets were HES (69.77%), SAL (62.27%) and ONS (53.28%). The CPRD Ethnicity Records, launched in June 2023, were not used in any published papers by December 2024; however, by this date, they were approved for 112 protocols and released for 47.

**Table 2:** CPRD dataset utilisation in papers published in 2016–2024, stratified by primary care data source

Papers published in 2016–2024	CPRD GOLD only (%)	CPRD GOLD & CPRD aurum (%)	CPRD Aurum only (%)	Total (%)
All	1772 (78.10%)	317 (13.97%)	180 (7.93%)	2269 (100.00%)
Without linked/CPRD algorithm-derived datasets	405 (17.85%)	27 (1.19%)	16 (0.71%)	448 (19.74%)
With linked dataset				
HES (any)	1176 (51.83%)	261 (11.50%)	146 (6.43%)	1583 (69.77%)
SAL (any)	1042 (45.92%)	231 (10.18%)	140 (6.17%)	1413 (62.27%)
ONS	900 (39.67%)	218 (9.61%)	91 (4.01%)	1209 (53.28%)
NCRAS	116 (5.11%)	13 (0.57%)	4 (0.18%)	133 (5.86%)
MINAP	34 (1.50%)	4 (0.18%)	0 (0.00%)	38 (1.67%)
MHDS/MHSDS	18 (0.79%)	8 (0.35%)	0 (0.00%)	26 (1.15%)
COVID-19 (any)	0 (0.00%)	6 (0.26%)	4 (0.18%)	10 (0.44%)
With CPRD Algorithm-derived dataset				
CPRD mother-baby link	54 (2.38%)	11 (0.48%)	6 (0.26%)	71 (3.13%)
CPRD pregnancy register	48 (2.12%)	16 (0.71%)	5 (0.22%)	69 (3.04%)
CPRD ethnicity record	0 (0.00%)	0 (0.00%)	0 (0.00%)	0 (0.00%)

Table 3 shows the cumulative utilisation of linked datasets and CPRD algorithm-derived datasets stratified by CPRD primary care data sources used in papers published between 2016 and 2024. While 15.29% of the CPRD protocols relied on a single linked dataset, 64.74% used multiple linked datasets: three were used in 39.67% of the cases, followed by two (20.10%), four (4.94%), and five, used only by one protocol. Only 2.20% of the CPRD protocols utilised a CPRD algorithm-derived dataset, and 1.98% used two.

**Table 3:** Cumulative utilisation of linked and CPRD algorithm-derived datasets stratified by CPRD primary care data source

Number	CPRD GOLD Only (%)	CPRD GOLD & CPRD Aurum (%)	CPRD Aurum Only (%)	Total (%)
CPRD linkages				
0	410 (18.07%)	27 (1.19%)	16 (0.71%)	453 (19.96%)
1	264 (11.64%)	51 (2.25%)	32 (1.41%)	347 (15.29%)
2	351 (15.47%)	55 (2.42%)	50 (2.20%)	456 (20.10%)
3	668 (29.44%)	157 (6.92%)	75 (3.31%)	900 (39.67%)
4	79 (3.48%)	26 (1.15%)	7 (0.31%)	112 (4.94%)
5	0 (0.00%)	1 (0.04%)	0 (0.00%)	1 (0.04%)
CPRD algorithm-derived datasets				
0	1,699 (74.88%)	301 (13.27%)	174 (7.67%)	2174 (95.81%)
1	44 (1.94%)	5 (0.22%)	1 (0.04%)	50 (2.20%)
2	29 (1.28%)	11 (0.48%)	5 (0.22%)	45 (1.98%)

Only 90 papers (3.97%) were associated with a CPRD protocol that used both linked and CPRD algorithm-derived datasets, and only five articles utilised solely the latter, as Table 4 shows:

**Table 4:** Utilisation of linked and/or CPRD algorithm-derived datasets stratified by CPRD primary care data source

Linked datasets	CPRD algorithm-derived datasets	CPRD GOLD only (%)	CPRD GOLD & CPRD aurum (%)	CPRD aurum only (%)	Total (%)
No	No	405 (17.85%)	27 (1.19%)	16 (0.71%)	448 (19.74%)
No	Yes	5 (0.22%)	0 (0.00%)	0 (0.00%)	5 (0.22%)
Yes	No	1294 (57.03%)	274 (12.08%)	158 (6.96%)	1726 (76.07%)
Yes	Yes	68 (3.00%)	16 (0.71%)	6 (0.26%)	90 (3.97%)

**Fig. 11:**
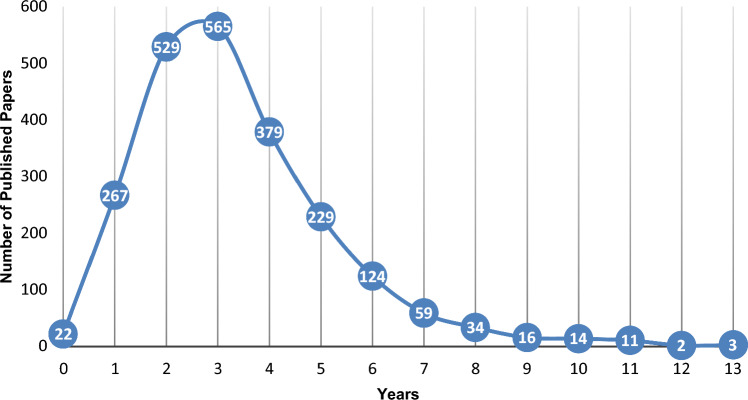
Time between CPRD protocol’s approval and publication for papers published in 2016–2024

For the papers published between 2016 and 2024, the time between their CPRD protocol’s approval and publication varied widely, as Fig. 11 reports. The most frequent time was three years (25.07%), followed by two years (23.47%) and four years (16.81%), while 21.83% of the articles took five or more years, up to 13, to be published.

**Fig. 12:**
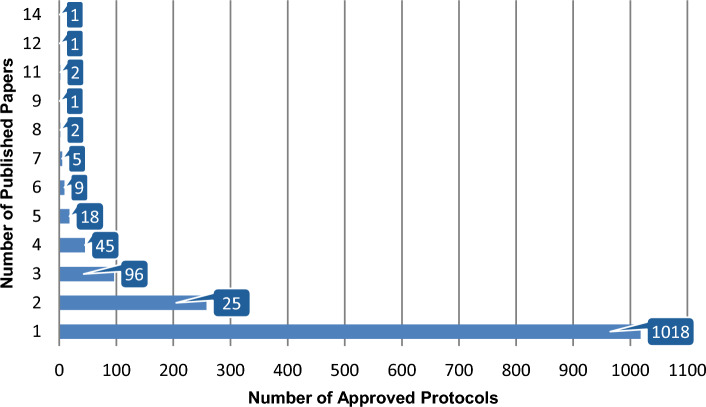
Published papers in 2016–2024 per CPRD approved protocol

The number of papers published between 2016 and 2024 per approved CPRD protocol varied, with the large majority of protocols being associated with one article only (69.92%) or two (17.72%), but with 2.68% supporting five or more, up to 14 papers (Fig. 12).

A small number of manuscripts (n = 46) were associated with more than one CPRD approved protocol, with the majority being associated with two (n = 42) up to five. In these cases, we calculated the CPRD protocol approval date as the earliest of all associated CPRD protocols’ approval dates.

### CPRD NICE guidelines papers

We found six CPRD papers that discussed NICE guidelines and their effects. One study investigated the impact of changes made to the NICE guidelines in 2002 on the treatment of rheumatoid arthritis [20]. Two studies investigated the impact of changes made to the NICE guidelines in 2004–2005 on antidepressant prescribing in adults [21] and children [22]. One study investigated the impact of changes made to the NICE guidelines in 2005 on referrals for suspected cancer [23]. Two studies investigated the impact of changes made to the NICE guidelines on type 2 diabetes management in 2009 [24] and in 2015 [25].

The CPRD papers reporting on NICE guidelines can be identified by the ‘*nice’* flag set to 1 in the *cprd_biblio_full.csv* file available in the Supplementary Material (Appendix 1).

### CPRD OMOP CDM papers

We found 43 papers in the CPRD eligible bibliography that used CPRD data standardised to the OMOP CDM, with a steep increase in recent years. While from 2014 to 2018 we only found one paper per year, we counted 18 papers in 2024.

**Fig. 13:**
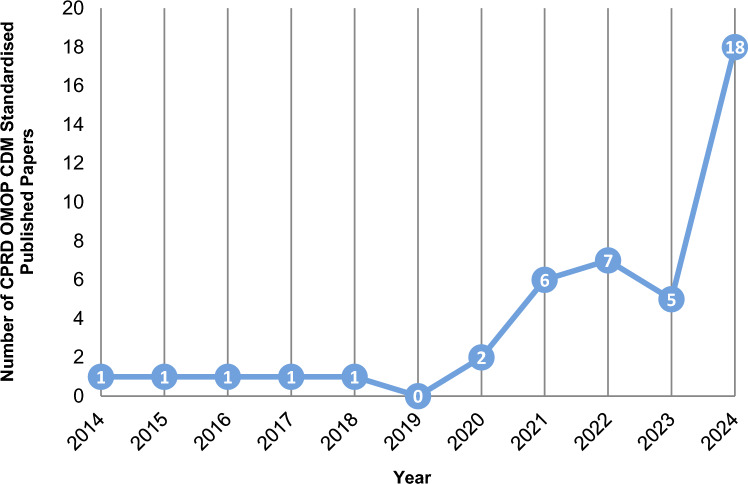
Papers using CPRD data standardised to OMOP Common Data Model (CDM) in 1988–2024

Figure 13 below shows this trend. The CPRD papers using data in OMOP CDM format can be identified by the ‘*omop’* flag set to 1 in the *cprd_biblio_full.csv* file available in the Supplementary Material (Appendix 1).

## Discussion and conclusion

We conducted an in-depth analysis of the CPRD research productivity and scientific impact between 1988 and 2024, including 3779 peer-reviewed articles covering more than 35 years of national and international clinical research. Using both Scopus and WoS scientific databases and searching for DOIs, titles and PMIDs with a three-step algorithm, we retrieved enriched metadata for 98.78% of the official CPRD bibliography up to December 2024. Our results confirmed a growing research productivity and consequent scientific impact based on CPRD data sources. The most prolific countries publishing studies utilising CPRD data were the UK (61.72%), the US (12.81%), and Canada (7.04%). The three most frequently represented institutional affiliations among authors were ‘*McGill University*’, the ‘*University of Manchester*’, and the ‘*University of Oxford*’, with seven UK universities ranking among the top ten most productive institutions. The most active journals publishing CPRD-related research have been ‘*BMJ Open*’, ‘*Pharmacoepidemiology and Drug Safety*’, and the ‘*British Journal of General Practice*’.

This study was the first to explore the utilisation of CPRD’s data sources by using the relationship between CPRD protocols and the manuscripts based on them published between 2016 and 2024. We also looked at each primary care dataset, linked dataset and CPRD algorithm-derived dataset individually, and in combination. This investigation provided a detailed and informative map of how CPRD’s data sources were used by researchers, and the information retrieved is available in the Supplementary Material (Appendix 1). Our results showed that 86.35% of articles employed CPRD GOLD exclusively, 8.39% used both CPRD GOLD and CPRD Aurum, and 4.76% utilised CPRD Aurum alone. Since 2020, there has been a decline in the exclusive use of CPRD GOLD, accompanied by an increased adoption of CPRD Aurum, either independently or in conjunction with CPRD GOLD.

Moreover, our findings provided evidence of the pivotal and valuable role of data linkages, as 80.26% of the CPRD bibliography published between 2016 and 2024 used linked or CPRD algorithm-derived data. The three most frequently linked datasets were Hospital Episode Statistics (69.77%), Small Area Linkages (62.27%) and Office for National Statistics mortality (53.28%), while 64.74% of the manuscripts used multiple linked datasets.

Finally, we reviewed the CPRD publications that explored the effect of NICE guidelines on clinical practice. These studies provide valuable real-world evidence on how guidance changes affect clinical practice. We also identified those manuscripts that used the OMOP CDM to run network studies to inform regulatory decision-making and reported a clear increase in the utilisation of OMOP CDM since 2020.

The information on CPRD data sources used in publications based on their protocol could be slightly inaccurate because protocols can be amended over time, and additional data sources can be added or removed. For example, it is likely that a proportion of the articles reported to have used both CPRD GOLD and CPRD Aurum might, in fact, have relied solely on CPRD GOLD, especially those papers published shortly after the launch of CPRD Aurum. Moreover, when multiple papers were published based on one single protocol, not all of them necessarily used every data source requested in the associated protocol: rather, each article drew only on the data relevant to its reported analyses. However, considering the substantial number of papers, a manual classification was not feasible, and that approach as well would have been error-prone without at least two researchers reviewing each paper.

It is also important to note that additional papers may exist beyond those included in the official CPRD Bibliography: in fact, some publications might be difficult to retrieve, especially if they have access restrictions, and/or if the authors did not inform CPRD about their publication.

Finally, a small number of papers currently classified as published in 2024 might be moved at a point by the publishers to 2025.

## Supplementary Information

Below is the link to the electronic supplementary material.

Supplementary file1 (DOCX 15 KB)

Supplementary file2 (CSV 1123 KB)

Supplementary file3 (BIB 15168 KB)

Supplementary file4 (BIB 18 KB)

Supplementary file5 (BIB 171 KB)

Supplementary file6 (BIB 25378 KB)

Supplementary file7 (BIB 40 KB)

Supplementary file8 (BIB 386 KB)

Supplementary file9 (XLSX 557 KB)

## Data Availability

The CPRD bibliography is publicly available, and the datasets used in this study are provided as Supplementary Material, including the data we curated to establish the links between CPRD protocols and manuscripts published between 2016 and 2024 (Appendix 1).
