# Supplementary material for: Academic impact and research data utilisation of the clinical practice research datalink: scientometric analyses
Source: Eur J Epidemiol. 2026 Jan 24;41(2):245–60. doi: 10.1007/s10654-025-01347-1 (PMC12975798; doi:10.1007/s10654-025-01347-1)
Supplement: Supplementary file 1 — Supplementary file1 (DOCX 15 KB) [file 10654_2025_1347_MOESM1_ESM.docx]

### Supplementary Material

1. *cprd_biblio_full.csv file contains the CPRD bibliography used in this manuscript (‘year’, ‘authors’, ‘title’, ‘journal, ‘doi/url’), with the ‘protocol_id’ and ‘approval_date’, and flags for primary care data (‘gold’, ‘aurum’), linked data (‘hes’, ‘ons’, 'sal’, ‘ncras’, ‘minap’, ‘mhds_mhsds’, ‘covid’), CPRD algorithm-derived (‘mother_baby’, ‘pregnancy’, ‘ethnicity’) data, OMOP CDM data standardisation (‘omop’) and NICE guidelines (‘nice’).*
2. *cprd_biblio_scopus_wos.zip file includes six bib files as produced by SCOPUS and WoS:*

- scopus_found_doi_cleaned.bib
- scopus_found_title_cleaned.bib
- scopus_found_pmid_cleaned.bib
- wos_found_doi_cleaned.bib
- wos_found_title_cleaned.bib
- wos_found_pmid_cleaned.bib

1. *cprd_biblio_supplementary.xlsx* contains several tabs as follows:

- cprd_bibliography_query: conditions used in Ovid Medline to identify papers missing from the original CPRD bibliography.
- cprd_datasource_categories: categorisation of all CPRD data sources.
- cord_research_productivity: numbers of CPRD papers and growth by year.
- cprd_frequent_journals_overall: number of CPRD papers by journal.
- cprd_top10_journals_overtime: number of CPRD papers among the top 10 most frequent journals over time.
- cprd_authors: number of journal articles by author.
- cprd_citations_top20authors: citation metrics (h, g and m index) from the top 20 most frequently published authors.
- cprd_contributing_countries: number of articles by country involving CPRD data, and by single or multiple country publications.
- cprd_affiliations: number and frequency of affiliations of all co-authors in papers using CPRD data.
- cprd_papers_citations: number of citations by publication using CPRD data.
- cprd_annual_top_keyword: top one annual keyword in articles using in CPRD data, based on authors keywords and Keywords Plus from Scopus and Web of Science.
- cprd_paper_category: categorisation for CPRD papers without a CPRD protocol.
- cprd_primarycare_utilisation: number of paper using CPRD GOLD and/or CPRD Aurum data by year.
